# Hybridization of cultivated *Vitis vinifera* with wild *V. californica* and *V. girdiana* in California

**DOI:** 10.1002/ece3.1797

**Published:** 2015-11-19

**Authors:** Gerald S. Dangl, Mary Lou Mendum, Judy Yang, M. Andrew Walker, John E. Preece

**Affiliations:** ^1^ Foundation Plant ServicesUniversity of California DavisOne Shields Ave.DavisCalifornia95616; ^2^ Department of Plant SciencesUniversity of California DavisOne Shields Ave.DavisCalifornia95616; ^3^ Department of Viticulture and EnologyUniversity of California DavisOne Shields Ave.DavisCalifornia95616; ^4^ National Clonal Germplasm RepositoryUSDA‐ARSUniversity of California DavisOne Shields AveDavisCalifornia95616

**Keywords:** California wild grape, domesticated plan introduction, genetic diversity, mixed‐species ancestry, natural hybrids, plant conservation genetics

## Abstract

Hybridization of introduced domesticates and closely related natives is well documented in annual crops. The widespread introduction of the domesticated grapevine, *Vitis vinifera,* into California where it overlaps with two native congenerics, with which it is interfertile, provides opportunity to investigate hybridization between woody perennials. Although geographically widespread, the introduction over the past two centuries has been limited to a few elite clonal cultivars, providing a unique opportunity to study the effects of hybridization on the native species. The amount of hybridization with *V*.* vinifera* and the genetic diversity of wild‐growing *Vitis californica* and *Vitis girdiana* were examined using nineteen microsatellite markers. STRUCTURE analysis was used to define hybrid and introgressed individuals and to analyze genetic structure of the native species. FAMOZ software was used to identify which *V. vinifera* cultivars served as parents of *F*_1_ hybrids. The three species were clearly distinguished by STRUCTURE analysis. Thirty percent of 119 *V*.* californica* vines were hybrids. The domesticated parent was identified for 16 *F*_1_ hybrid vines; the original California cultivar, ‘Mission’, was the parent of eight. Backcrosses were also found, showing introgression into subsequent generations. Similar results were obtained for a small sample of *V. girdiana*. Removing hybrids greatly reduced the genetic variation of the presumed pure species, among which there was essentially no genetic structure. Limited genetic variability indicates the California natives may be threatened by genetic erosion. The discovery of *F*_1_ hybrids of ‘Mission’, a cultivar not grown in the areas for ~100 years, suggests long generation times for wild vines that, often, grow into expansive liana and propagate by layering, all factors that limit recruitment in populations already disjunct by habitat lose. Hermaphroditic flowers and fruit that is more attractive to birds may favor the production of backcross seed and establishment of introgressed individuals.

## Introduction

Anthropomorphic introduction of species beyond their native range is an increasingly frequent occurrence, as the movement of people and commercial goods becomes more global and transportation more rapid. The introduction of non‐native species can damage ecosystems and endanger native species (Allendorf et al. 2001). Allopatric congenerics often lack genetic reproductive barriers and will produce hybrids when they become sympatric. Hybridization between introduced domesticated species and native congeners may be particularly problematic (Ellstrand et al. 1999; Ayres et al. 2004). The introduction of a crop species may involve the sudden establishment of many individuals often over large areas with the introduced domestic benefiting from human assistance over many years of cultivation. The world's most important crops are annuals. Hybridization and introgression of important annual crops, such as bean, maize, rice, and wheat with their wild relatives and the consequences for the natives are well documented (Andersson and de Vicente 2010; Ellstrand et al. 2013). The effects of hybridization and introgression of introduced woody perennials with their wild relatives are not as well studied (Nassar 2002; Meirmans et al. 2010).

The genus *Vitis* comprises ~60–70 species of dioecious woody vines with wind and insect‐borne pollen. Most grape species grow in the understory of riparian ecosystems in the northern hemisphere (Levadoux et al. 1962). *Vitis* displays the classic distribution of a Tertiary relic genus, with *V. sylvestris* in Europe, clusters of species in East Asia, eastern North America, and western North America, and a few species in Central America (Milne and Abbott 2002; Péros et al. 2011). Species are maintained primarily through physical isolation by distance or geographic barriers (i.e., allopatry) and, to a lesser extent, by phenology. When in proximity and with sufficient overlap in bloom time, *Vitis* species readily hybridize, a trait long exploited by breeders to produce hybrid rootstock and scion cultivars with resistance to pathogens, environmental stresses, and diseases (Mullins et al. 1992).

The best‐known member of the genus is the cultivated grapevine, *V. vinifera* ssp. *vinifera* (*V. vinifera*)*,* which was domesticated from the wild European grape, *V. vinifera* ssp. *sylvestris* (*V. sylvestris*), although the location and number of domestication events are still under active debate (Aradhya et al. 2003; Arroyo‐García et al. 2006; Riaz et al. 2013). The selection and subsequent vegetative propagation of mutant hermaphrodite vines was a primary factor in the domestication of *V. vinifera* (Aradhya et al. 2003). Wide‐scale commercial production for wine, juice, fresh fruit, and raisins has taken *V. vinifera* well beyond its original native range; it is now grown on all continents except Antarctica.

In California, there are two endemic *Vitis* species: *V. girdiana* in the south and *V. californica* in the northern Central Valley, with occasional natural hybridization between them where they are sympatric (Olmo and Koyama 1980). The two species differ in leaf shape, berry size, seed morphology, and the degree of tomentum on their leaves and shoots (Wada and Walker 2012). Both species are found in riparian habitats. *Vitis girdiana* is found in or near springs and creeks from Baja California to the Tehachapi Mountains and from coastal areas to the desert regions of California and southern Nevada. *Vitis californica* is found from the Tehachapi Mountains in the south to southern Oregon and is common in the Central Valley and scattered to about 1000 m in the Coastal Range, Sierra Nevada, Cascade, and Klamath Mountains.

*Vitis vinifera* came initially to southern California with Spanish missionaries starting in 1769 and expanded north as new missions were built (Wagner 1974). For ~100 years, the introduction was limited to a single cultivar, ‘Listán Prieto’. Of Spanish origin, this ancient cultivar had a long history of cultivation in South America under the name ‘Criolla Chica’ (Tapia et al. 2007). It was so central to the missionary way of life that in California this cultivar became known simply as ‘Mission’. It is extremely hardy, fast growing, high yielding, and well adapted to California's Mediterranean climate (McKee 1947). The 1849 Gold Rush and resulting settlement spread ‘Mission’ across much of *V. californica* and *V. girdiana's* native ranges, creating a prolonged introduction of a single genetic individual. The expansion of the California wine industry in the late 1800s and early 1900s brought a limited number of new European grape cultivars to California.

Knowledge of the amount and distribution of genetic variation of *V. californica* and *V. girdiana* and the degree of admixture with domesticated *V. vinifera* is central to conservation efforts aimed at preserving the native species. Here, we use microsatellite markers to document hybridization of *V. californica,* and *V. girdiana,* with each other and with the domesticated *V. vinifera*. We address these specific questions. Can first‐generation hybrids (*F*_1_) be verified by identifying the domesticated parent from among the limited number of *V. vinifera* cultivars historically and currently grown in California? Given that nearly all *V. vinifera* cultivars are hermaphrodites, do a portion of hybrids inherit this trait? Do later‐generation backcrosses survive in the wild and can they be distinguished from the *F*_1_ generation using microsatellite markers? Can wild‐growing vines with admixture be differentiated from those without admixture? What is the genetic variation that exists among wild‐growing, pure, native *V. californica,* and *V. girdiana* germplasm? Finally, we examined the unique history and nature of the introduction of *V. vinifera*, which for a century consisted of a single genetic individual and since then has consisted of several dozen cultivars. This unique introduction is traced, and its implications for conservation are discussed.

## Material and Methods

### Plant materials

The study set included 119 unique genotypes from wild‐collected vines presumed to be *V. californica* (CAL), 26 genotypes from wild‐collected vines presumed to be *V. girdiana* (GRD), and 45 diverse *V. vinifera* cultivars (VIN) that included most cultivars of current or historic importance in California (Table S1). Wild vines were selected based on location and leaf morphology. All 26 GRD genotypes and 53 of the CAL genotypes were from vines maintained in the vineyard of the Department of Viticulture and Enology, University of California, Davis. These vines were collected from various locations in California covering the ranges of the two species as part of a previously published study (Wada 2008). An additional 31 unique CAL genotypes were from wild vines collected in the Napa Valley wine‐producing region of California, close to commercial vineyards (Klaassen et al. 2011). The remaining 34 CAL genotypes were collected specifically for this study. Twenty‐five were collected from remote areas of Shasta County in northern California, at least 45 km from extant commercial vineyards. Nine vines were collected from Yolo County, within 10 km of extant vineyards. In the previous studies cited above, some wild vines growing as far as 200 m apart, typically following creeks, shared identical profiles; therefore, we sampled from vines that were growing at least 400 m apart to prevent repeat sampling of natural clones. As a reference, we included the ornamental cultivar ‘Roger's Red’, a known *V. californica* × *V. vinifera* hybrid, originally collected in Napa Valley (Dangl et al. 2010).

### DNA extraction, amplification, and fragment sizing

Collected samples consisted of young, fresh, green leaves that were dried using chemical desiccants (Bautista et al. 2008). Whole genomic DNA was extracted using a DNeasy Plant Mini Kit (Qiagen, Valencia, CA). The 190 unique genotypes representing three *Vitis* species were defined by 19 microsatellite marker loci: VVMD5 and 7 (Bowers et al. 1996), VVMD21, 24, 25, 27, 31, and 32 (Bowers et al. 1999), VrZAG62, 79, and 93 (Sefc et al. 1999), VVS2 (Thomas and Scott 1993), UDV108 and 124 (Di Gaspero et al. 2005), VVIP26 (Merdinoglu et al. 2005), VMCNG3a10 (Riaz et al. 2007), VMC7f2 (Pellerone et al. 2001), and VMC5a10 and 8g9, which are available on the Italian *Vitis* Database (http://www.vitisdb.it). The VrZAG series was derived from the North American species *V. riparia*; the rest were originally cloned from *V. vinifera*. Two additional markers were used for flower sex determination: VVIb23 (Fechter et al. 2012) and APT3 (Battilana et al. 2013).

PCR was conducted in a total volume of 10 *μ*L containing 5 ng genomic DNA and 1X Gold Buffer, 2 mmol/L MgCl_2_, 0.8 mmol/L of each dNTP, 0.13 units AmpliTaq Gold DNA polymerase, and 2 pmol of each primer (all from Applied Biosystems, Foster City, CA). Forward primers were labeled with one of three fluorescent dyes: 6‐FAM, HEX, or NED. The thermal‐cycler regime was 5 min at 94°C, followed by 30 cycles of 30 sec at 94°C, 1 min at 54°C, and 1 min at 72°C, concluding with 1 cycle of 7 min at 72°C. To generate microsatellite profiles, 0.5–0.8 *μ*L of each of three amplified products was multiplexed using fluorescent dye and mixed with 10 *μ*L formamide and 0.25 *μ*L GeneScan 400HD ROX size standard (Applied Biosystems). Samples were denatured at 94°C for 5 min prior to electrophoresis on an ABI Prism 3130 × 1 Genetic Analyzer (Applied Biosystems) through a 36‐cm capillary array with POP7 as the matrix. Allele binning, based on estimated size in base pairs (bp), and label editing were performed using GenoTyper 2.5 software (Applied Biosystems). When the template DNA for a given individual failed to amplify at a particular locus after four attempts, it was scored as homozygous for a single null allele.

### Genetic diversity

The uniqueness of all 190 genotypes was confirmed, and the polymorphic information content of each locus was calculated (Botstein et al. 1980) using the Microsatellite Toolkit (Park 2001). The probability of identity was calculated using the FAMOZ software package (Gerber et al. 2003). For each of the 19 microsatellite loci, the number of alleles, allele frequencies, observed and expected heterozygosity, and the fixation index were calculated using GenAlEx 6.0 (Peakall and Smouse 2006). Allelic richness was calculated in FSTAT (Goudet 2002), which applies rarefaction for comparison of different sample sizes (El Mousadik and Petit 1996).

### Analysis of population structure

Model‐based Bayesian analysis implemented in the software package STRUCTURE (Pritchard et al. 2000) was used to determine the approximate number of genetic clusters (*K*) within the full data set and to assign individuals to the most appropriate cluster. All simulations were run using the assumptions that individuals may have admixed ancestry and that allele frequencies are correlated (Falush et al. 2003). Simulations were run varying *K* as a prior from one to ten. After multiple trials, a burn‐in of 80,000 iterations and 100,000 iterations for data collection proved sufficient to produce results that were consistent among eight runs for likely values of *K*. The most likely value for *K* was determined based on averages of the estimated Ln probability of the data (ln Pr(*X*/*K*) as described in the STRUCTURE documentation and by calculating ∆*K* (Evanno et al. 2005). Bar graphs from STRUCTURE were prepared using STRUCTURE PLOT (Ramasamy et al. 2014). STRUCTURE was also used to generate the posterior probability that individuals have mixed ancestry (the “GENSBACK” option with, *K* = 3 and *M* = 0.05). For this analysis, assignment to one of the three sample groups was given as a prior. The results indicate whether an individual has mixed ancestry within the three preceding generations (G = 3) or if the individual is best assigned to another sample group.

Additional tests to investigate possible cryptic structure within the CAL samples were performed with 50,000 iterations burn‐in and 250,000 iterations for data collection; eight runs for each value of *K* from 1 to 7 were simulated. As before, admixed ancestry and correlated allele frequencies were assumed. To facilitate visualization of these results, most CAL samples were placed in one of three subgroups based on collection location. The “Wine Country” subgroup contained 37 samples primarily from Napa County, with a few from the adjacent counties of Lake, Solano, and Yolo. The 35 samples in the “Remote” subgroup were primarily from Shasta County, with a few samples from the adjacent counties of Siskiyou and Tehama. The third subgroup, “Other”, contained samples from scattered locations throughout the range of *V. californica*. This location information was not used as a prior for STRUCTURE analysis. The proportion of each individual attributed to each inferred cluster (*Q*) was averaged over the eight runs. Genetic structure within and among the Wine Country and Remote subgroups was also investigated using PCoA (principal coordinate analysis) computed in GenAlX, using the codominant genotypic distance of Smouse and Peakall (1999).

### Detection of parent–progeny pairs

The FAMOZ software package (Gerber et al. 2003) was used to determine whether any of the wild‐collected CAL and GRD vines had *V. vinifera* cultivars from the study set as a parent. Single‐parent cumulated exclusion probabilities and the single‐parent LOD score (the logarithm of the likelihood odds ratio) were calculated based on 16 microsatellite loci. Two mismatching loci were allowed. We also used simple exclusion to eliminate possible parents from a database of over 1200 unique genotypes of *V. vinifera* cultivars and hybrid rootstock cultivars. This analysis used eight loci that maximized overlap with the database.

## Results

### Allelic variation at 19 microsatellite loci

The combined 19 microsatellite markers uniquely distinguished all 190 sampled vines. Samples collected specifically for this study were from vines growing at least 400 m apart; there were no duplicate profiles among them based on the 19 markers, indicating that 400 m of separation was sufficient to avoid repeated sampling of one individual spread through natural clonal propagation. The cumulative “probability of identity”, a measure of the likelihood that two individuals randomly share an identical profile, reached less than one in a billion with only nine loci for the 119 CAL samples, with six loci for the 26 GRD samples, and with five loci for the 45 VIN samples (data not shown).

Despite sample size differences, the number of alleles (Na) was similar for the VIN and CAL groups and slightly lower for the GRD group (Table 1). However, allelic richness (Rs) in the VIN group was greater than in the CAL group at 16 of 19 loci and the PIC (polymorphic information content) was greater in VIN than in CAL at 16 loci. Over all 19 markers, both Rs and PIC were lower in CAL and GRD groups than in VIN group. Although the GRD group had far fewer samples and a lower Na than the CAL group, the Rs and PIC were greater in GRD than in CAL (Table 1).

**Table 1:** Allelic diversity at 19 microsatellite loci for 190 vines that ostensibly represent three Vitis species. *Vitis vinifera* (VIN) is represented by 45 common diverse cultivars, the *V. californica* (CAL) set contains 119 vines collected in wild settings, and *V. girdiana* (GRD) contains 26 wild‐collected vines

Locus	Number of alleles			Allelic richness			Polymorphic information content		
	VIN	CAL	GIRD	VIN	CAL	GIRD	VIN	CAL	GIRD
VrZAG93	7	10	7	6.0	5.1	6.8	0.57	0.53	0.66
VrZAG79^a^	10	8	5	9.6	6.4	4.9	0.85	0.66	0.43
VVMD27^a^	8	10	10	7.6	5.1	9.8	0.80	0.44	0.74
VVMD21	6	10	5	5.9	7.1	5.0	0.62	0.68	0.61
VrZAG62^a^	8	9	5	7.3	5.6	4.9	0.74	0.51	0.49
VVMD25	6	10	5	5.3	5.9	4.8	0.72	0.34	0.24
VVS2^a^	11	9	9	9.6	4.7	9.0	0.81	0.20	0.76
VMC8g9	13	13	8	11.7	7.0	8.0	0.89	0.42	0.78
UDV124	11	17	7	10.2	8.8	6.6	0.84	0.60	0.49
VVMD24	6	9	5	5.9	5.1	4.9	0.63	0.39	0.37
VVIP26	8	10	7	6.9	5.7	6.8	0.78	0.52	0.64
VVMD5^a^	8	15	6	7.8	8.0	5.8	0.82	0.61	0.64
VMC7f2	6	5	3	5.4	2.2	3.0	0.49	0.06	0.29
UDV108	10	8	10	8.5	4.9	9.6	0.77	0.46	0.67
VMCNG3a10	11	11	7	9.6	5.8	6.9	0.82	0.28	0.61
VMC5a10	5	7	7	4.5	4.0	6.5	0.64	0.35	0.56
VVMD7^b^	10	8	4	8.8	5.6	3.8	0.71	0.56	0.27
VVMD31^b^	7	7	5	6.5	5.3	5.0	0.74	0.41	0.50
VVMD32^b^	8	8	5	7.9	4.2	5.0	0.80	0.27	0.50
Range	5–13	5–13	4–10	4.5–11.7	2.2–8.8	3.8–9.8	0.5–0.9	0.1–0.7	0.2–0.8
Mean	8.37	9.68	6.32	7.63	5.60	6.16	0.74	0.44	0.54

a Eight loci included to maximize overlap with reference databases (see This et al. 2004).

b At these three markers, multiple individuals failed to produce any amplified fragment. All such samples were recorded as being homozygous for a single null allele. (At VVMD7, 20% and at VVMD31, 74% of CAL individuals failed to produce an amplified fragment. At VVMD32, 65% of GIRD individuals failed amplify.

Two loci failed to amplify fragments of any size in multiple samples of the CAL group; 23 samples (20%) failed to amplify at VVMD7 and 88 (74%) failed at VVMD31. At VVMD32, seventeen GRD samples (65%) also failed to amplify. As the same DNA extractions readily produced fragments at all other loci, these nonamplifying sample–locus combinations were scored as homozygous for a single null allele for Table 1 (also see Table S2). These three problematic loci were omitted from subsequent statistics and analyses, although their use as diagnostic markers is discussed below.

### Diversity within and among three grape species‐based groups

The 16 remaining loci were used to calculate averages for several measures of diversity (Table 2). The cultivated VIN group had higher allelic richness than the other groups, a higher observed and expected heterozygosity (Ho and He), and a fixation index (*F*) close to zero. The cultivars in the VIN group have little in common except that they are or were historically grown in California. In contrast, the nondomesticated GRD and CAL groups both had a positive *F*‐value. The positive *F*‐value for the GRD group (0.25) results from a significant dearth of heterozygotes (*P *<* *0.0001). Although the averaged Ho and He were not significantly different within the larger CAL group, He was higher than Ho at each of the 16 markers, resulting in an *F*‐value of 0.105.

**Table 2:** Genetic diversity of three species‐based sample groups averaged over 16 microsatellite marker loci. Results are also presented for two subgroups of the 119 wild‐collected *Vitis californica* vines and the 26 wild‐collected *V. girdiana* vines. The subgroups consist of individuals determined to be hybrids and the remaining individuals designated as pure; see text

Population	Sample size	Na	Rs	Ho	Ho SE	He	He SE	*F*
*V. vinifera*	45	8.38	4.37	0.762	0.038	0.764	0.026	0.004
*V. girdiana*	26	6.60	3.34	0.443	0.037	0.599	0.043	0.250
Pure *V. girdiana*	21	4.53	2.95	0.385	0.044	0.539	0.052	0.302
Hybrid *V. girdiana*	5	4.73	4.34	0.690	0.054	0.692	0.028	−0.008
*V. californica*	119	10.06	2.71	0.416	0.035	0.475	0.046	0.105
Pure *V. californica*	84	4.88	2.07	0.297	0.037	0.363	0.052	0.175
Hybrid *V. californica*	35	8.69	3.84	0.698	0.050	0.668	0.041	−0.053

Na, average number of alleles; Rs, average allelic richness; Ho, average observed heterozygosity; He, average expected heterozygosity; *F*, fixation index; SE, standard error.

### Genetic structure among sample groups

**Figure 1:**
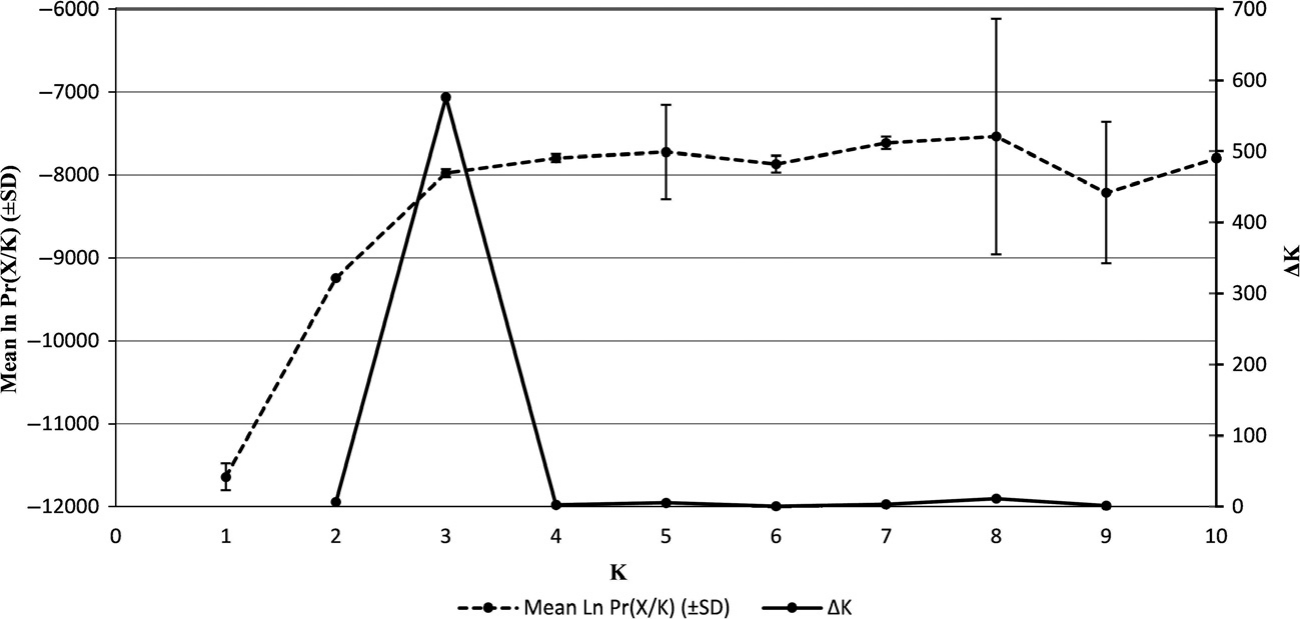
The approximate number of genetic clusters (*K*) within the full data set of 190 individuals based on results from the software package STRUCTURE. The “estimated log probability of the data”, Ln Pr(*X*/*K*), (Pritchard et al. 2000), and Δ*K* (Evanno et al. 2005) are shown for each value of *K* from one to ten. Results are derived from eight separate simulations for each value of *K*. Both methods show strong support for *K* being equal to three, consistent with the three species in the data set.

Based on both the estimated log probability of the data (ln Pr(*X/K*), Pritchard et al. 2000) and ∆*K* (Evanno et al. 2005), the most likely number of genetic clusters in the entire 190‐sample data set is three, as expected for samples from three distinct species (Fig. 1).

**Figure 2:**
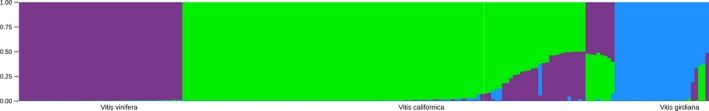
Bar graph of the estimated membership coefficient, *Q*, for each of the 190 individuals in each of three genetic clusters (*K*). The most likely value of *K* inferred by STRUCTURE was three (see Fig. 1). Each genotype is represented by a vertical bar the colored segments represent the proportion of *Q* in each of the three clusters. Within each of the three species‐based groups, individuals were sorted for decreasing values of *Q* for the genetic cluster to which the majority of the group was assigned. Data are an average over eight runs.

When the individual 190 samples were arranged according to their estimated degree of membership (*Q*) in each of the three clusters, an interesting picture emerged (Fig. 2). The 45 *V. vinifera* cultivars comprise one clearly defined cluster. None show any introgression from *V. californica* or *V. girdiana*. These European cultivars have long histories with most predating European settlement in California, or they were derived from crosses between such parents. The cultivated grape is assumed to have been domesticated from the wild form, *V. sylvestris*, in the Near East (McGovern et al. 1996). Separated by an ocean, a continent, and millennia, it is not surprising that these cultivars show no introgression from *V. californica* or *V. girdiana*.

The CAL and GRD sample groups also formed well‐defined clusters, but both clearly contained individuals of admixed ancestry, either with *V. vinifera* or with each other (Fig. 2). Twenty‐one of the 26 GRD samples had *Q* > 0.95 for the *V. girdiana* cluster (*Q*_GRD_). The five remaining samples had *Q*_GRD_ values between 0.52 and 0.82. For two of these samples, the next highest proportion is from *V. californica*, and for the remaining three samples, the admixture came from *V. vinifera*. There were also individuals of clearly mixed ancestry among the 119 CAL samples. The estimated membership coefficient in the CAL cluster (*Q*_CAL_) was below 0.9 for 33 of the CAL samples. Among these samples, the vast majority of the non‐CAL identity came from *V. vinifera*, but three had contribution from *V. girdiana* with *Q*_GRD_ values above 0.1, one as high as 0.321 (Fig. 2).

### Identification of individuals with mixed‐species ancestry

**Figure 3:**
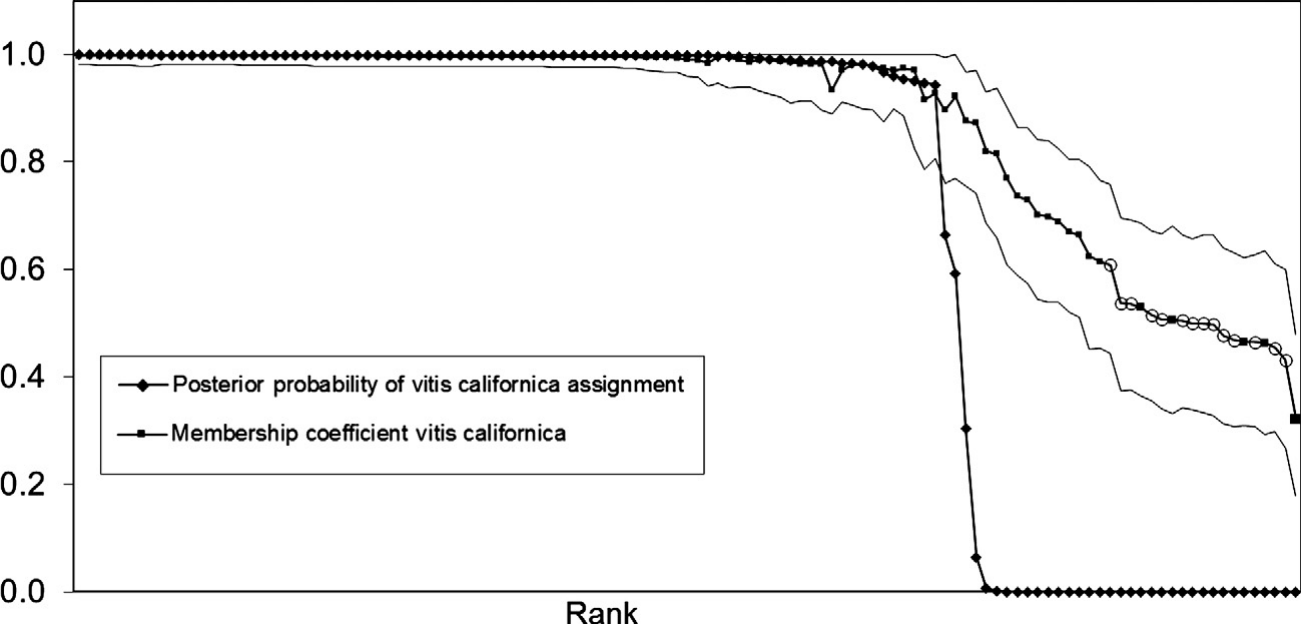
The posterior probability of correct assignment to the CAL cluster (*P*_CAL_) for each of the 119 vines collected as wild *Vitis californica* is presented with the estimated membership coefficient to the CAL cluster (*Q*_CAL_). Samples are ranked by decreasing *P*_CAL_. For both measures, the number of possible clusters was fixed at 3 (*K* = 3). *P*_CAL_ was calculated in STRUCTURE using the “GENSBACK” option with the species groups given as a prior. The *Q*_CAL_ values are shown bounded by the upper and lower ends of the 90% probability interval. These are the same *Q*_CAL_ values used to generate Figure 2. Open circles denote the 14 *F*_1_ hybrids with for which the *V. vinifera* parent could be determined.

Prior to examining the genetic variability within native California grapes, it was necessary to determine which samples represent the true variation of the species and which are hybrids. Based on a *Q* value of 0.90 and above as a demarcation, 86 CAL samples and 21 GRD samples could be considered “pure” natives. The GENSBACK option within STRUCTURE provides an additional method to delineate wild‐growing hybrids or backcrossed individuals from individuals without apparent admixture. With the number of populations (*K* = 3) and population assignment of each individual (CAL, GRD or VIN) provided as priors, GENSBACK runs simulations then calculates the posterior probability (*P*) that an individual has the correct population assignment, that an individual is from a population different than the one assigned or has recent ancestry in a different population. In Figure 3, the 119 CAL samples are ranked by the posterior probability that each individual was correctly assigned to the *V*. *californica* cluster (*P*_CAL_), which is shown superimposed on *Q*_CAL_. The complimentary results show 84 CAL samples with *P*_CAL_ of 0.94 or higher and *Q*_CAL_ of 0.92 or higher (Fig. 3). Consistent with results ex infra, these individuals were deemed “pure” *V. californica* (pure CAL). Two marginal individuals with *Q*_CAL_ of ~0.90 had *P*_CAL_ values of 0.66 and 0.59 (much lower than the next highest value of 0.94). These two anomalous individuals were placed with the other admixed individuals. Thus, 35 of the 119 wild‐collected *V. californica* vines (29%) were classified as hybrids (CAL hybrids).

The demarcation was more definitive for the 26 GIRD samples. Twenty‐one samples had *P*_GRD_ and *Q*_GRD_ above 0.95. For the remaining five samples, *P*_GRD_ was essentially zero and the highest *Q*_GRD_ was 0.81 (Table S3). The clear distinction between pure *V. girdiana* and hybrids may be due to the small sample size.

### Lineage of the hybrids

For an individual, that is, not correctly assigned, GENSBACK option generates the posterior probabilities (*P*) that the individual is best assigned to a different cluster or has recent ancestry from a different cluster. We examined the previous three generations (G = 3). The analysis showed that the majority of the 35 CAL hybrids have recent ancestry from the *V. vinifera* cluster (Table 3). Four CAL hybrids (1–4 in Table 3) have the highest probability of actually being best assigned to the *V. vinifera* cluster. The GENSBACK results must be considered in context and cannot be strictly interpreted. The analysis assigns probabilities to one of the a priori groups. The apparently incongruent assignment for these four samples is due to the presence of many alleles from *V. vinifera* ancestors and a few alleles that are high frequency in both the VIN and CAL set. In context, these four are not escaped *V. vinifera* cultivars; they do not match any known cultivar. Nor are they *V. vinifera* × *V. vinifera* seedlings, all four have alleles exclusive to *V. californica*. The first sample is most likely a *V. vinifera* × *V. californica* hybrid backcrossed to *V. vinifera*. The remaining three samples, as well as samples five through twenty, are likely first‐generation hybrids (*F*_1_ hybrids). Ten of the remaining CAL hybrids (samples 21–30) had *P* indicating more distant *V. vinifera* ancestry; these individuals are backcrosses to *V. californica* for one or two generations. Samples 31, 32, and 33 in Table 3 appear to be *V. californica* × *V. girdiana* hybrids, perhaps backcrossed to *V. girdiana*. For the final two samples, the highest probability was assignment to the *V. californica* cluster (*P*_CAL_ values of 0.66 and 0.59). These were the anomalous individuals that formed our hybrid cut‐off.

**Table 3:** Posterior probability of immigrant ancestry for 35 *V. californica* vines determined to be hybrids, see text. The probabilities indicate whether an individual is from a population different than the one assigned or has recent ancestry from a different population. Collection location relative to known recent or current grape production and the name of the cultivated parent, where such could be determined, are also shown. Bold font highlights the largest portion of probability

	Origin	Known parent	Probability of *Vitis vinifera* assignment or ancestry				Probability of *Vitis girdiana* assignment or ancestry			
			Assignment	1st generation	2nd generation	3rd generation	Assignment	1st generation	2nd generation	3rd generation
1	Wine Country		**1.00**	0	0	0	0	0	0	0
2	Remote	Mission	**0.89**	0.11	0	0	0	0	0	0
3	Remote	Mission	**0.79**	0.21	0	0	0	0	0	0
4	Remote		**0.72**	0	0.09	0.20	0	0	0	0
5	Remote	Mission	0	**1.00**	0	0	0	0	0	0
6	Wine Country	Alicante Bouschet	0	**1.00**	0	0	0	0	0	0
7	Wine Country	Malbec	0	**1.00**	0	0	0	0	0	0
8	Remote	Mission	0	**1.00**	0	0	0	0	0	0
9	Wine Country	Merlot	0	**0.98**	0.02	0	0	0	0	0
10	Wine Country		0.02	**0.98**	0.01	0	0	0	0	0
11	Wine Country	Saint George	0	**0.97**	0.03	0	0	0	0	0
12	Wine Country	Cabernet Sauvignon	0.04	**0.95**	0.01	0	0	0	0	0
13	Wine Country	Cabernet Sauvignon	0	**0.93**	0.07	0	0	0	0	0
14	Remote	Mission	0.38	**0.62**	0	0	0	0	0	0
15	Remote	Mission	0.39	**0.61**	0	0	0	0	0	0
16	Wine Country	Cabernet Sauvignon	0.43	**0.57**	0	0	0	0	0	0
17	Remote	Mission	0.44	**0.56**	0	0	0	0	0	0
18	Wine Country	Zinfandel	0.47	**0.52**	0.01	0	0	0	0	0
19	Wine Country		0.50	**0.50**	0	0	0	0	0	0
20	Remote	Mission	0.47	**0.49**	0.04	0	0	0	0	0
21	Wine Country		0	0.01	**0.96**	0.03	0	0	0	0
22	Remote		0	0	**0.95**	0.05	0	0	0	0
23	Remote		0	0	**0.83**	0.16	0	0	0	0
24	Remote		0	0	**0.82**	0.18	0	0	0	0
25	Remote		0	0	**0.75**	0.25	0	0	0	0
26	Remote		0.09	0	**0.71**	0.21	0	0	0	0
27	Remote		0	0	**0.66**	0.34	0	0	0	0
28	Remote		0.41	0.09	**0.48**	0.01	0	0	0	0
29	Remote		0.04	0	0.10	**0.85**	0	0	0	0
30	Remote		0	0	0.37	**0.63**	0	0	0	0
31	Remote^a^	Ramsey	0	0.26	0.20	0.01	0	0.01	**0.47**	0.05
32	Remote		0	0	0	0	0	0	0.11	**0.83**
33	Remote		0	0	0	0	0	0	0.02	**0.67**
34	Remote^b^		0	0	0.02	0.38	0	0	0	0.00
35	Wine Country^b^		0	0	0	0.06	0	0	0.02	0.25

a This vine was collected at the Whiskeytown Lake Visitors center. Although at least 15 km from commercial vineyards, a vine of the rootstock ‘Ramsey’ was found at this location.

b The greatest portion of probability for these 2 vines was assignment to the *Vitis californica* cluster (0.59 and 0.66).

Five of the *V. girdiana* samples showed contributions from other clusters. Two of the hybrids appeared to have *V. californica* ancestry; the remaining three were hybrids with *V. vinifera* (Table S3).

Given the limited number of clonal cultivars introduced into the native ranges of *V. californica* and *V. girdiana*, it should be possible to identify the cultivated parent of first‐generation hybrids. The FAMOZ software employs likelihood analysis methods to find parent, progeny triads, and pairs from sets of microsatellite data. It identified *V*. *vinifera* parents for 14 of the 35 CAL hybrids. Eight of these 14 *F*_1_ hybrids were from crosses with ‘Mission’, three with ‘Cabernet Sauvignon’, and one each with ‘Merlot’, ‘Zinfandel’, and ‘Alicante Bouschet’ (Table 3). The single‐parent exclusion probability was 0.998 for 16 loci. Three CAL hybrids (samples 31, 32, and 33 in Table 3) appeared to be “*V. californica* × *V. girdiana*”. However, sample 31, and several apparent “*V. californica* × *V. vinifera*” hybrids, had alleles not found in the rest of the entire study set, suggesting rare native alleles or contributions from additional *Vitis* species. Simple exclusion analysis identified the rootstock ‘Ramsey’, a natural hybrid of *V. candicans* × *V. rupestris* from Texas, as a parent for the apparent “*V. californica* × *V. girdiana*” hybrid sample 31, and the once‐common *V. rupestris* rootstock ‘Saint George’ as a parent for one apparent “*V. californica* × *V. vinifera*” hybrid (sample 11 in Table 3). Of the five samples classified as *V. girdiana* hybrids, one was a ‘Mission’ *F*_1_ hybrid, two appeared to be *V. vinifera* backcrosses, and two appeared to be *V. californica* hybrids (Table S3).

### Diagnostic markers

At the locus VVMD7, 82 of the 84 wild *V. californica* individuals classified as “pure”, ex supra, either failed to amplify any fragment or appeared homozygous for the 241‐bp allele, which has not been observed in *V. vinifera* (Laucou et al. 2011). Consistent with the assumption that any wild‐collected *V. californica* with an allele other than 241 bp at VVMD7 is potentially a hybrid, all but one of the thirty‐five CAL hybrids were either heterozygous for the *V. californica* exclusive 241‐bp allele and a *V. vinifera* allele or “homozygous” for a *V. vinifera* allele; the assumption being these are heterozygous with the high‐frequency *V. californica* null allele; the one exception was a hybrid with the common rootstock ‘Saint George’ (Table S2). The results were more definitive at VVMD31, where all but one of the 84 pure CAL failed to amplify and all 16 confirmed *F*_1_ crosses were “homozygous” for the alleles found in their non‐*californica* parent. Any presumed *V. californica* vine that amplifies a fragment of any size at VVMD31 is either a hybrid or has a potentially interesting, very low‐frequency *V. californica* allele (Table S2).

The marker VVMD32 may be useful as a diagnostic marker for *V. girdiana*, although confirmation requires more samples to be analyzed. Seventeen of the 21 pure *V. girdiana* failed to amplify a fragment of any size; the remaining four pure *V. girdiana* were homozygous for the 245‐bp allele, which is not found in the other two species. All five of the hybrid *V. girdiana* were homozygous for alleles also found in *V. vinifera* or *V. californica*.

### Sex determination of pure and admixed *V. californica* samples

*Vitis californica* vines are dioecious, as are most Vitis species. Imperfect flowers are a requisite for true, pure native germplasm. Sex determination in *Vitis* is controlled by a single locus with three alleles; the hermaphrodite allele (H) is dominant over the female allele (*F*) with the male allele (M) dominant over both H and F (Antcliff 1980). Male vines are MF; female vines are FF. The selection and subsequent vegetative propagation of mutant hermaphrodite vines was a primary factor in the domestication of *V. vinifera*. Nearly all cultivars are hermaphrodites, primarily heterozygotes (HF), although there are a few HH hermaphrodites as well (e.g., ‘Chardonnay’ and ‘Riesling’). A subset of the 119 CAL individuals was tested using DNA markers to determine their sex and to see how these results compared with our distinction between native and introgressed *V. californica* vines. Of the eight confirmed *F*_1_ hybrids tested, five were female (FF) and three hermaphroditic (HF); there were no males. These results are consistent with hermaphroditic *V. vinifera* (HF or HH) pollen donors fertilizing female (FF) wild *V. californica*. If these *F*_1_'s were backcrossed by wild male (MF) *V. californica*, half of the progeny would be male; the other half would be female (FF) or hermaphrodites (HF or HH), depending on the *F*_1_. A mix of female and hermaphrodites would also result from a wild female (FF) being pollinated by a hermaphrodite (HF or HH) *F*_1_. Consistent with these expectations, of the ten presumed backcrosses tested, four were male, five were female, and one was a hermaphrodite. In contrast, among the 66 pure CAL samples tested, there were 49 males, 27 females, and no hermaphrodites. Finding only males and females in our pure individuals does not prove these vines are free of *V. vinifera* introgression, all three flower types are possible results of backcrosses, but imperfect flowers are a requisite for true *V. californica*.

### Variability among pure *V. californica*

Removing the 35 admixed individuals from the 119 samples in the original CAL group greatly reduced the allelic variability among the remaining samples. The average number of alleles dropped from 10.06 for the full CAL set to 4.88 for the 84 pure CAL samples, while Ne dropped from 2.14 to 1.74 (Table 2). Thus, a large portion of the low‐frequency alleles among the original CAL group came from *V. vinifera* and other species involved in generating the hybrids. The limited polymorphism among the 84 pure CAL was characterized by one or two very high‐frequency alleles at most loci. At four of 16 loci, one allele was essentially fixed (frequency above 0.9); at an additional nine loci, only two alleles combined for a frequency over 0.90.

### Structure among the 84 pure *V. californica* samples

The 84 pure CAL individuals with no identifiable introgression were assigned to three subgroups based on their location of collection: the “Wine Country” subgroup contained 37 individuals collected in Napa and adjacent counties, the “Remote” subgroup contained 35 individuals primarily from Shasta County with a few from neighboring counties, and the remaining 12 “Other” individuals were collected at various locations. From the STRUCTURE analysis, both the plateauing of *Q* and Δ*K* methods agreed that the most likely value of *K* is three. However, at *K* = 3 the Δ*K* was only 3.85; the next highest was 2.65, and the lowest was 2.07 at *K* = 2 and *K* = 6, respectively. At *K* = 3, there was a tendency for individuals from the same collection subgroup to be assigned primarily to the same cluster (Fig. S1). The 37 individuals in the “Wine Country” subgroup had an average *Q* of 0.4 for cluster 1 and 0.3 for the other two clusters. All six “Wine Country” individuals with a single‐cluster *Q* > 0.5 were assigned to cluster 1. The average *Q* for the 35 individuals of the “Remote” subgroup was 0.41 for cluster 3 and 0.29 for cluster 1. Six of the 35 “Remote” vines had *Q* > 0.5 for cluster 3, none were over 0.5 for cluster 1. As expected, the 12 “Other” individuals from random locations showed no particular affinity for either the “Wine Country” or “Remote” subgroups.

**Figure 4:**
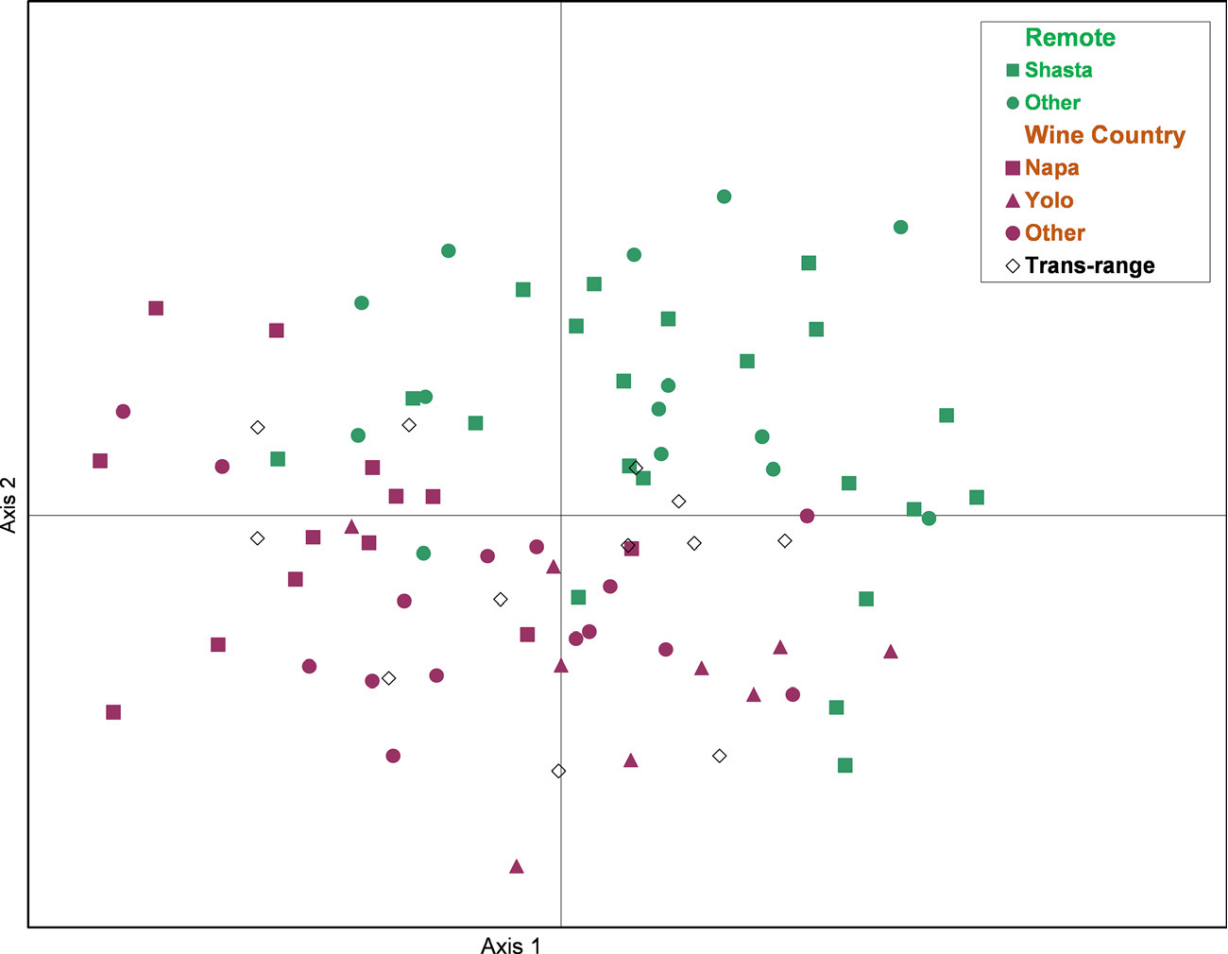
Principal coordinate analysis of the 84 “pure” *Vitis californica* genotypes based on 16 microsatellite markers. The 84 “pure” *V. californica* individuals are labeled based on where they were collected. The 35 vines collected away from extant vineyards (Remote, green) primarily clustered in the upper‐right quadrant. These samples are shown divided into a subset of vines collected in Shasta County within 32 km of each other, green squares, and those collected in other remote areas in Northern California, green circles. The 37 “Wine Country” samples, shown divided into subsets from Napa County (red squares), Yolo County (red triangles), primarily clustered in the lower quadrants. Twelve individuals collected at various other locations across the range of *V. californica* (white diamonds), do not cluster.

Principal coordinate analysis was used to confirm the limited genetic variability of the pure CAL samples detected by STRUCTURE and to provide an additional means to visualize the results. The first coordinate accounted for only 25% of the variation and the second accounted for 19%, confirming the limited genetic variability among the 84 pure CAL vines. However, the “Remote” individuals dominated in the upper‐right quadrant and the “Wine Country” individuals dominated the lower left quadrant, although there was an overlap between these subgroups in the other two quadrants (Fig. 4). We further highlighted three subsets of individuals. The first subset contained 21 “Remote” samples collected within 32 km of each other in Shasta County over 200 km north of the other two subsets. The second subset contained 13 “Wine Country” individuals collected within 20 km of each other in the Napa Valley. The third subset of eight “Wine Country” individuals was collected in Yolo County along Cache Creek, which is separated from the Napa Valley by a mountain range. The Napa and Yolo subsets did separate along the first coordinate, suggesting weak structure in *V. californica* (Fig. 4).

## Discussion

The establishment of *V. vinifera* cultivars into the native ranges of *V. californica* and *V. girdiana* is powered by the sheer number of new establishment sites, vineyards, and the incursion of these vineyards further into remote areas of the native ranges. The cultivars themselves do not constitute a genetic invasion. We found no evidence of *V. vinifera* establishing outside of cultivation, although one sample was potentially a *V. californica* × *V. vinifera* backcross to *V. vinifera*. Under cultivation, *V. vinifera* seed production is primarily through selfing and results in very high inbreeding depression; such seeds produce very few normal, vital seedlings.

We did find that introgression of *V. vinifera* alleles into *V. californica* is pervasive; the same is likely true for *V. girdiana*. Introgression of *V. vinifera* alleles may place the natives in danger of genetic swamping. We identified first‐generation *V. californica* × *V. vinifera* and *V. girdiana* × *V. vinifera* hybrids, and we document *V. californica* × *V. vinifera* backcrosses. Genetic erosion may also threaten the native populations as their habitat is lost or diminished, reducing the number of individuals and further isolating the naturally somewhat disjunct native populations.

The results presented here confirm that *V. californica* and *V. girdiana* are distinct species, settling a century‐old disagreement in the literature. Ravaz (1902) considered *V. girdiana* to be a collection of hybrids between *V. californica* and *V. vinifera*. Munson (1909) stated that *V. girdiana* had a sufficiently unique appearance and habitat to be considered a separate species. Modern taxonomists side with Munson and treat *V. californica* and *V. girdiana* as separate species (Wada and Walker 2012). Our data and other recent work (Wada 2008) provide further molecular confirmation. It is possible that the vines on which Ravaz based his conclusion were, in fact, hybrids.

Eight of the wild‐collected *V. californica* were *F*_1_ hybrids, with the cultivar ‘Mission’ as the *V. vinifera* parent. All eight were collected in remote, apparently undisturbed, natural habitats. We also found one *V. girdiana* × *V. vinifera* cv. ‘Mission’ hybrid. ‘Mission’ came into the areas where these hybrids were collected with the gold rush of 1849. It has not been widely grown in northern California for ~100 years, suggesting vines in these habitats may be very long‐lived. A more diverse set of *V. vinifera* parents was found for *F*_1_ wild vines growing near extant vineyards. These parents were more recently introduced cultivars; their hybrid offspring are almost certainly younger than the ‘Mission’ *F*_1_ vines, demonstrating that hybridization is ongoing and will continue wherever the two species become sympatric.

In a European study of gene flow from cultivated to wild grape, one hybrid of a common rootstock and *V. sylvestris* was detected (Di Vecchi‐Staraz et al. 2009). Our finding of two *F*_1_ hybrids of *V. californica* and rootstock cultivars shows that, when sympatric, hybridization between *V. californica* and grape rootstocks will occur and that the *F*_1_ hybrids can establish. Genetic transformation of rootstocks is being considered as a strategy for managing specific diseases and stresses. Genetic transformation of rootstocks as opposed to scions may be a means of avoiding consumers’ trepidations regarding “genetically modified organisms”. It would also reduce the risk of the transgenes escaping; in commercial vineyards, rootstocks are not allowed to produce shoots and flower. However, vineyards planted on transgenic rootstock would need to be properly tended to prevent rootstock suckers from producing flowers. The risk of transgene escape would increase if such vineyards were abandoned. The risk would also be much greater near plantings of transgenic rootstock mother vines at grapevine nurseries.

We found strong evidence for introgression of *V. vinifera* alleles into *V. californica* and *V. girdiana* beyond first‐generation hybrids. Given our small sample sizes, finding multigenerational introgression suggests some *V. vinifera* traits are advantageous within the ranges of *V. californica* and *V. girdiana*. Hybrids and backcrosses may produce more seed and more attractive fruit, favoring seed dispersal by birds. Hermaphroditic flowers, which were critical in the domestication of *V. vinifera* from its wild progenitor (Arroyo‐García et al. 2006), is one *V. vinifera* trait likely to benefit hybrids and backcrosses. It is hard to imagine the relatively infrequent native vines successfully pollinating the self‐compatible hermaphroditic *V. vinifera* cultivars in a vineyard. We can surmise *V. vinifera* acts almost exclusively as the pollen donor in the spontaneous hybrid crosses. As such, half of the *F*_1_ generation will be hermaphrodites. Heterosis may free the *F*_1_ hybrids from the high inbreeding depression that limits production of *V. vinifera* self‐seedlings. If so, selfing of the *F*_1_ generation could establish the *F*_2_ generation, a large percentage of which will also carry the hermaphrodite allele. In this work, we could not assign a *V. vinifera* parent to several CAL samples that had *Q*_CAL_ and *P*_CAL_ values expected of an *F*_1_. These individuals may in fact be *F*_2_ *V. californica* × *V. vinifera* hybrids.

There was no substantive genetic structure among the 84 *V. californica* individuals designated as “pure”. This low variation could be attributed to the markers employed. However, these loci show much greater variation in *V. vinifera* and other Vitis species (Aradhya et al. 2013; Riaz et al. 2013). Wild populations of *V. sylvestris* in Europe experienced extreme pressures from rapid habitat loss combined with the devastating impact of introduced fungal disease and phylloxera. Although genetic diversity measures of *V. sylvestris* populations are low compared to *V. vinifera* cultivars (Di Vecchi‐Staraz et al. 2009; Lopes et al. 2009), they are higher than those we found for *V. californica*. Six microsatellite loci not used in this study also had one allele with a frequency over 0.9 within a set of 36 distinct genotypes from wild‐collected *V. californica* vines (Klaassen et al. 2011); these vines would be classified as pure by the criteria set forth in this work. The limited variation in *V. californica*, consistent over 25 microsatellite loci, appears to be genuine.

The limited variation suggests genetically pure *V. californica* is threatened by genetic erosion. The genus *Vitis* has the distribution of a Tertiary relic. *Vitis californica* has the northern‐most range among the west coast *Vitis* species and thus presumably travelled farthest from the ancestral refugium as the glaciers retreated. The low genetic variability among *V. californica* may be the result of one or more genetic bottlenecks (Milne and Abbott 2002; Péros et al. 2011). Our finding of first‐generation *V. californica* × ‘Mission’ hybrids indicates a long generation time of wild‐growing vines. In their natural habitat, individual *V. californica* vines will grow into extensive lianas with a proclivity for natural clonal propagation. The resulting low light within the understory disadvantages seedling establishment. These factors, combined with a paucity of female vines as seen in this study and consistent with years of field observations, can severely limit recruitment and encourage inbreeding.

The long generation time also provides ample opportunity for *F*_1_ hybrids to backcross to native *V. californica* and *V. girdiana,* fostering the production of backcross seed at the expense of conspecific seed. We found ten individuals that appeared to be later‐generation *V. californica* × *V. vinifera* backcrosses (21–30 in Table 3), exposing *V. californica* populations to the possibility of genetic swamping. The situation is likely similar for *V. girdiana*.

Genotyping wild populations and rouging all but pure *V. californica* and *V. girdiana* could be an effective, if impractical, means to preserve the species. Alternatively, conservation efforts for *V. californica* and *V. girdiana* should start by preventing the deliberate introduction of non‐native *Vitis* species into remote native habitat. For instance, we found the rootstock ‘Ramsey’ and one of its *V. californica* hybrid offspring growing outside of the visitor's center at the Whiskeytown National Recreation Area in Shasta County. The parent vine was almost certainly deliberately planted as landscaping, introducing an unnecessary source of hybrids into a prime *V. californica* habitat.

Riparian restoration and maintenance conservation efforts in Northern California require a readily available source of *V. californica* vines, from defined regions, that have been tested and confirmed not to be hybrids. The diagnostic and sex markers developed here could be particularly useful to quickly eliminate hybrids and backcrosses. Vines being grown for restoration purposes could be tested during the nursery stage, or propagated from an already well‐characterized collection maintained at a suitable facility such as that of the USDA/ARS National Clonal Germplasm Repository in Davis. Such a collection would need to represent what diversity still exists in the native species and to have an appropriate mix of male and female vines. The apparent limited variability of *V. californica* means candidates for such a collection cannot be selected based solely on DNA marker analysis. Candidate vines should be collected from diverse habitats, including soil types, and from remote areas on the edges of the species range. Genetic analysis of additional samples, perhaps with additional microsatellite markers, may aid in identifying a mix of vines that preserves existing genetic variation and maintains an appropriate mix of male and female vines.

## Conflict of Interest

None declared.

## Supporting information


**Figure S1.** The estimated membership coefficient (Q) for 84 *Vitis californica* individuals in each of three genetic clusters (*K*).

**Table S1.** Alphabetical list of the 45 *Vitis vinifera* cultivars used as references in some analyses.

**Table S2.** Allele frequencies for 190 unique genotypes representing three *Vitis* species at 19 microsatellite markers.

**Table S3.** Estimated membership coefficient and posterior probabilities of correct assignment and immigrant ancestry for 26 wild‐collected *Vitis girdiana* vines.
