# Supplementary figures and images for: Hybridization of cultivated Vitis vinifera with wild V. californica and V. girdiana in California
Source: Ecol Evol. 2015 Nov 19;5(23):5671–84. doi: 10.1002/ece3.1797 (PMC4813103; doi:10.1002/ece3.1797)

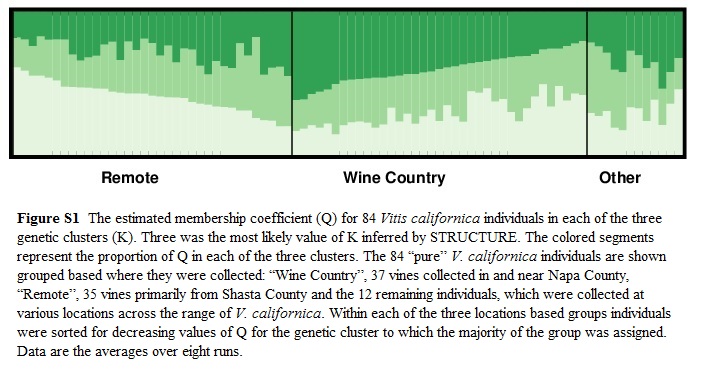

Supplement: Supplementary file 1 — Figure S1. The estimated membership coefficient (Q) for 84 Vitis californica individuals in each of three genetic clusters (K). [file ECE3-5-5671-s001.jpg]
