# Supplementary material for: Hybridization of cultivated Vitis vinifera with wild V. californica and V. girdiana in California
Source: Ecol Evol. 2015 Nov 19;5(23):5671–84. doi: 10.1002/ece3.1797 (PMC4813103; doi:10.1002/ece3.1797)
Supplement: Supplementary file 2 — Table S1. Alphabetical list of the 45 Vitis vinifera cultivars used as references in some analyses. [file ECE3-5-5671-s002.docx]

| **Table S1** Alphabetical list of the 45 *Vitis vinifera* cultivars used as references in some analyses. Cultivars were selected to represent *V. vinifera* diversity and include most cultivars of current or historical importance in California. The genotypes for these cultivars are derived from vines maintained by Foundation Plant Services, U.C. Davis. | | |
| --- | --- | --- |
| Aglianico | Chenin blanc | Rhazaki Arhanon |
| Alicante Bouschet | Counoise | Riesling |
| Aleatico | Dolcetto | Riesling italico |
| Aligote | Grenache | Ruby Cabernet |
| Aramon noir | Macabeo | Sangiovese |
| Assyrtico blanc | Malbec | Sauvignon vert |
| Barbera | Malvasia bianca | Savagnin blanc |
| Blauer Portugieser | Mavrodaphne | Sylvaner blanc |
| Burger | Melon | Tannat |
| Cabernet Franc | Merlot | Teroldego |
| Cabernet Sauvignon | Mission | Trincadeira |
| Carignane | Muscat Of Alexandria | Touriga Nacional |
| Carmenère | Muscat Ottonel | Trebbiano Toscano |
| Chardonnay | Pedro Ximenez | Thompson Seedless |
| Chasselas blanc | Pinot noir | Zinfandel |
