# Supplementary material for: Hybridization of cultivated Vitis vinifera with wild V. californica and V. girdiana in California
Source: Ecol Evol. 2015 Nov 19;5(23):5671–84. doi: 10.1002/ece3.1797 (PMC4813103; doi:10.1002/ece3.1797)
Supplement: Supplementary file 3 — Table S2. Allele frequencies for 190 unique genotypes representing three Vitis species at 19 microsatellite markers. [file ECE3-5-5671-s003.docx]

| **Table S2** Allele frequencies for 190 unique genotypes representing three *Vitis* species at 19 microsatellite markers. Pure and Hybrid are subsets of the Wild collected *V. californica* and *V. girdiana* groups. Vine template DNA and marker combinations that repeatedly failed to produce any amplified fragment were assumed to be homozygous for a single null allele (Null X2). | | | | | | | | |
| --- | --- | --- | --- | --- | --- | --- | --- | --- |
| **Locus** | **Allele** | ***V. vinifera* n=45** | **Wild *V. californica* n=119** | **Pure *V. californica* n=84** | **Hybrid *V. californica* n=35** | **Wild *V. girdiana* n=26** | **Pure *V. girdiana* n=21** | **Hybrid *V. girdiana* n=5** |
| **VrZAG93** | **189** | 0.589 | 0.009 | 0 | 0.029 | 0 | 0 | 0 |
|  | **191** | 0.056 | 0 | 0 | 0 | 0 | 0 | 0 |
|  | **197** | 0.011 | 0 | 0 | 0 | 0 | 0 | 0 |
|  | **199** | 0.167 | 0.022 | 0 | 0.071 | 0 | 0 | 0 |
|  | **201** | 0 | 0.004 | 0 | 0.014 | 0.019 | 0.024 | 0 |
|  | **213** | 0 | 0.004 | 0.006 | 0 | 0 | 0 | 0 |
|  | **215** | 0.111 | 0.060 | 0.012 | 0.171 | 0.442 | 0.500 | 0.200 |
|  | **217** | 0.011 | 0 | 0 | 0 | 0 | 0 | 0 |
|  | **219** | 0 | 0.009 | 0.006 | 0.014 | 0.019 | 0 | 0.100 |
|  | **221** | 0 | 0 | 0 | 0 | 0.288 | 0.286 | 0.300 |
|  | **223** | 0 | 0.431 | 0.500 | 0.271 | 0.077 | 0.071 | 0.100 |
|  | **225** | 0 | 0 | 0 | 0 | 0.096 | 0.119 | 0 |
|  | **227** | 0 | 0.453 | 0.469 | 0.414 | 0.058 | 0 | 0.300 |
|  | **231** | 0.056 | 0.004 | 0 | 0.014 | 0 | 0 | 0 |
|  | **236** | 0 | 0.004 | 0.006 | 0 | 0 | 0 | 0 |
|  |  |  |  |  |  |  |  |  |
| **VrZAG79** | **237** | 0.022 | 0 | 0 | 0 | 0 | 0 | 0 |
|  | **239** | 0.033 | 0 | 0 | 0 | 0 | 0 | 0 |
|  | **243** | 0.167 | 0.022 | 0 | 0.071 | 0.021 | 0 | 0.100 |
|  | **245** | 0.111 | 0.259 | 0.278 | 0.214 | 0.708 | 0.789 | 0.400 |
|  | **247** | 0.144 | 0.022 | 0 | 0.071 | 0 | 0 | 0 |
|  | **249** | 0.056 | 0 | 0 | 0 | 0 | 0 | 0 |
|  | **251** | 0.189 | 0.026 | 0 | 0.086 | 0.125 | 0.105 | 0.200 |
|  | **255** | 0.044 | 0.190 | 0.210 | 0.143 | 0 | 0 | 0 |
|  | **257** | 0.067 | 0.440 | 0.475 | 0.357 | 0.125 | 0.079 | 0.300 |
|  | **259** | 0.167 | 0.022 | 0.006 | 0.057 | 0 | 0 | 0 |
|  | **261** | 0 | 0.022 | 0.031 | 0 | 0.021 | 0.026 | 0 |
|  |  |  |  |  |  |  |  |  |
| **VVMD27** | **175** | 0.044 | 0.004 | 0 | 0.015 | 0 | 0 | 0 |
|  | **179** | 0.144 | 0 | 0 | 0 | 0 | 0 | 0 |
|  | **181** | 0.178 | 0.004 | 0 | 0.015 | 0 | 0 | 0 |
|  | **183** | 0.033 | 0 | 0 | 0 | 0 | 0 | 0 |
|  | **185** | 0.167 | 0.030 | 0 | 0.103 | 0.021 | 0 | 0.100 |
|  | **189** | 0.267 | 0.151 | 0.140 | 0.176 | 0.042 | 0 | 0.200 |
|  | **191** | 0.022 | 0.707 | 0.774 | 0.544 | 0.021 | 0 | 0.100 |
|  | **193** | 0 | 0.082 | 0.079 | 0.088 | 0 | 0 | 0 |
|  | **194** | 0.144 | 0.009 | 0 | 0.029 | 0 | 0 | 0 |
|  | **199** | 0 | 0.004 | 0.006 | 0 | 0 | 0 | 0 |
|  | **203** | 0 | 0 | 0 | 0 | 0.146 | 0.184 | 0 |
|  | **205** | 0 | 0.004 | 0 | 0.015 | 0 | 0 | 0 |
|  | **213** | 0 | 0.004 | 0 | 0.015 | 0.021 | 0 | 0.100 |
|  | **215** | 0 | 0 | 0 | 0 | 0.313 | 0.368 | 0.100 |
|  | **219** | 0 | 0 | 0 | 0 | 0.083 | 0.079 | 0.100 |
|  | **221** | 0 | 0 | 0 | 0 | 0.313 | 0.316 | 0.300 |
|  | **223** | 0 | 0 | 0 | 0 | 0.021 | 0.026 | 0 |
|  | **225** | 0 | 0 | 0 | 0 | 0.021 | 0.026 | 0 |
|  |  |  |  |  |  |  |  |  |
| **VVMD21** | **217** | 0 | 0 | 0 | 0 | 0.021 | 0.025 | 0 |
|  | **221** | 0 | 0 | 0 | 0 | 0.333 | 0.325 | 0.375 |
|  | **223** | 0 | 0 | 0 | 0 | 0.042 | 0 | 0.250 |
|  | **229** | 0 | 0.004 | 0 | 0.014 | 0 | 0 | 0 |
|  | **231** | 0 | 0 | 0 | 0 | 0.438 | 0.450 | 0.375 |
|  | **239** | 0 | 0.004 | 0 | 0.014 | 0 | 0 | 0 |
|  | **241** | 0 | 0 | 0 | 0 | 0.167 | 0.200 | 0 |
|  | **243** | 0.222 | 0.043 | 0 | 0.143 | 0 | 0 | 0 |
|  | **247** | 0 | 0.274 | 0.305 | 0.200 | 0 | 0 | 0 |
|  | **249** | 0.522 | 0.038 | 0 | 0.129 | 0 | 0 | 0 |
|  | **254** | 0.044 | 0 | 0 | 0 | 0 | 0 | 0 |
|  | **255** | 0 | 0.013 | 0.018 | 0 | 0 | 0 | 0 |
|  | **256** | 0.078 | 0 | 0 | 0 | 0 | 0 | 0 |
|  | **258** | 0.067 | 0.026 | 0.006 | 0.071 | 0 | 0 | 0 |
|  | **266** | 0.067 | 0 | 0 | 0 | 0 | 0 | 0 |
|  | **270** | 0 | 0.150 | 0.159 | 0.129 | 0 | 0 | 0 |
|  | **271** | 0 | 0.423 | 0.476 | 0.300 | 0 | 0 | 0 |
|  | **273** | 0 | 0.026 | 0.037 | 0 | 0 | 0 | 0 |
|  |  |  |  |  |  |  |  |  |
| **VrZAG62** | **175** | 0 | 0.004 | 0 | 0.014 | 0 | 0 | 0 |
|  | **187** | 0.056 | 0 | 0 | 0 | 0 | 0 | 0 |
|  | **189** | 0.356 | 0.034 | 0 | 0.114 | 0 | 0 | 0 |
|  | **193** | 0 | 0.004 | 0 | 0.014 | 0 | 0 | 0 |
|  | **195** | 0.256 | 0.025 | 0 | 0.086 | 0 | 0 | 0 |
|  | **197** | 0.111 | 0.042 | 0 | 0.143 | 0.040 | 0 | 0.250 |
|  | **199** | 0.011 | 0.263 | 0.337 | 0.086 | 0 | 0 | 0 |
|  | **201** | 0.044 | 0.004 | 0 | 0.014 | 0.280 | 0.310 | 0.125 |
|  | **203** | 0.033 | 0.017 | 0.012 | 0.029 | 0.600 | 0.619 | 0.500 |
|  | **205** | 0.133 | 0 | 0 | 0 | 0.060 | 0.071 | 0 |
|  | **213** | 0 | 0.606 | 0.651 | 0.500 | 0.020 | 0 | 0.125 |
|  |  |  |  |  |  |  |  |  |
| **VVMD25** | **239** | 0 | 0.008 | 0.012 | 0 | 0 | 0 | 0 |
|  | **241** | 0 | 0.008 | 0 | 0.029 | 0 | 0 | 0 |
|  | **242** | 0 | 0.004 | 0 | 0.014 | 0 | 0 | 0 |
|  | **243** | 0.244 | 0.055 | 0.012 | 0.157 | 0.019 | 0 | 0.100 |
|  | **245** | 0.222 | 0.042 | 0 | 0.143 | 0.019 | 0 | 0.100 |
|  | **247** | 0 | 0.013 | 0 | 0.043 | 0.865 | 0.952 | 0.500 |
|  | **253** | 0.256 | 0.805 | 0.916 | 0.543 | 0.038 | 0 | 0.200 |
|  | **255** | 0 | 0.013 | 0.018 | 0 | 0 | 0 | 0 |
|  | **257** | 0 | 0.042 | 0.042 | 0.043 | 0 | 0 | 0 |
|  | **259** | 0.244 | 0.008 | 0 | 0.029 | 0 | 0 | 0 |
|  | **265** | 0 | 0 | 0 | 0 | 0.058 | 0.048 | 0.100 |
|  | **267** | 0.022 | 0 | 0 | 0 | 0 | 0 | 0 |
|  | **271** | 0.011 | 0 | 0 | 0 | 0 | 0 | 0 |
|  |  |  |  |  |  |  |  |  |
| **VVS2** | **123** | 0 | 0 | 0 | 0 | 0.043 | 0.026 | 0.125 |
|  | **133** | 0.278 | 0.038 | 0 | 0.132 | 0.022 | 0 | 0.125 |
|  | **135** | 0.067 | 0.889 | 0.970 | 0.691 | 0.022 | 0 | 0.125 |
|  | **137** | 0.067 | 0.009 | 0 | 0.029 | 0.065 | 0.079 | 0 |
|  | **139** | 0.067 | 0.009 | 0 | 0.029 | 0 | 0 | 0 |
|  | **143** | 0.200 | 0 | 0 | 0 | 0 | 0 | 0 |
|  | **145** | 0.067 | 0.013 | 0.006 | 0.029 | 0.370 | 0.368 | 0.375 |
|  | **147** | 0.022 | 0.004 | 0.006 | 0 | 0.022 | 0.026 | 0 |
|  | **149** | 0.011 | 0.017 | 0.012 | 0.029 | 0.130 | 0.158 | 0 |
|  | **151** | 0.178 | 0.013 | 0 | 0.044 | 0 | 0 | 0 |
|  | **153** | 0.011 | 0 | 0 | 0 | 0.152 | 0.158 | 0.125 |
|  | **155** | 0.033 | 0.009 | 0.006 | 0.015 | 0.174 | 0.184 | 0.125 |
|  |  |  |  |  |  |  |  |  |
| **VMC8g9** | **143** | 0 | 0.030 | 0.018 | 0.059 | 0 | 0 | 0 |
|  | **147** | 0 | 0.004 | 0.006 | 0 | 0.038 | 0.048 | 0 |
|  | **155** | 0 | 0.009 | 0.012 | 0 | 0 | 0 | 0 |
|  | **157** | 0 | 0.004 | 0.006 | 0 | 0 | 0 | 0 |
|  | **159** | 0.111 | 0.034 | 0 | 0.118 | 0.038 | 0 | 0.200 |
|  | **164** | 0.178 | 0 | 0 | 0 | 0.077 | 0.095 | 0 |
|  | **166** | 0 | 0.103 | 0.104 | 0.103 | 0 | 0 | 0 |
|  | **168** | 0.078 | 0.741 | 0.848 | 0.485 | 0.038 | 0 | 0.200 |
|  | **170** | 0.122 | 0 | 0 | 0 | 0 | 0 | 0 |
|  | **171** | 0.011 | 0.004 | 0 | 0.015 | 0 | 0 | 0 |
|  | **173** | 0.089 | 0.013 | 0 | 0.044 | 0 | 0 | 0 |
|  | **174** | 0.022 | 0 | 0 | 0 | 0.192 | 0.143 | 0.400 |
|  | **176** | 0.111 | 0.022 | 0 | 0.074 | 0.231 | 0.286 | 0 |
|  | **180** | 0.011 | 0 | 0 | 0 | 0 | 0 | 0 |
|  | **182** | 0 | 0.004 | 0.006 | 0 | 0.288 | 0.310 | 0.200 |
|  | **184** | 0 | 0.009 | 0 | 0.029 | 0.096 | 0.119 | 0 |
|  | **186** | 0.056 | 0 | 0 | 0 | 0 | 0 | 0 |
|  | **198** | 0.089 | 0.022 | 0 | 0.074 | 0 | 0 | 0 |
|  | **202** | 0.056 | 0 | 0 | 0 | 0 | 0 | 0 |
|  | **221** | 0.067 | 0 | 0 | 0 | 0 | 0 | 0 |
|  |  |  |  |  |  |  |  |  |
| **UDV124** | **188** | 0.033 | 0 | 0 | 0 | 0.038 | 0.048 | 0 |
|  | **194** | 0.022 | 0 | 0 | 0 | 0 | 0 | 0 |
|  | **198** | 0.133 | 0.014 | 0 | 0.050 | 0 | 0 | 0 |
|  | **206** | 0.022 | 0 | 0 | 0 | 0 | 0 | 0 |
|  | **208** | 0.200 | 0.060 | 0.019 | 0.167 | 0.019 | 0 | 0.100 |
|  | **210** | 0 | 0.005 | 0.006 | 0 | 0 | 0 | 0 |
|  | **212** | 0.078 | 0 | 0 | 0 | 0 | 0 | 0 |
|  | **214** | 0.044 | 0.032 | 0 | 0.117 | 0.019 | 0 | 0.100 |
|  | **216** | 0.078 | 0 | 0 | 0 | 0 | 0 | 0 |
|  | **218** | 0.033 | 0 | 0 | 0 | 0 | 0 | 0 |
|  | **220** | 0.122 | 0.197 | 0.184 | 0.233 | 0 | 0 | 0 |
|  | **221** | 0 | 0.005 | 0.006 | 0 | 0.058 | 0.048 | 0.100 |
|  | **223** | 0 | 0.005 | 0 | 0.017 | 0.192 | 0.167 | 0.300 |
|  | **228** | 0.233 | 0.014 | 0.006 | 0.033 | 0 | 0 | 0 |
|  | **232** | 0 | 0.028 | 0.025 | 0.033 | 0.654 | 0.714 | 0.400 |
|  | **234** | 0 | 0.009 | 0.013 | 0 | 0.019 | 0.024 | 0 |
|  | **242** | 0 | 0.005 | 0 | 0.017 | 0 | 0 | 0 |
|  | **258** | 0 | 0.005 | 0.006 | 0 | 0 | 0 | 0 |
|  | **263** | 0 | 0.014 | 0.019 | 0 | 0 | 0 | 0 |
|  | **265** | 0 | 0.573 | 0.665 | 0.333 | 0 | 0 | 0 |
|  | **266** | 0 | 0.023 | 0.032 | 0 | 0 | 0 | 0 |
|  | **267** | 0 | 0.009 | 0.013 | 0 | 0 | 0 | 0 |
|  | **271** | 0 | 0.005 | 0.006 | 0 | 0 | 0 | 0 |
|  |  |  |  |  |  |  |  |  |
| **VVMD24** | **204** | 0 | 0.750 | 0.837 | 0.543 | 0.167 | 0.125 | 0.375 |
|  | **206** | 0 | 0.140 | 0.163 | 0.086 | 0.750 | 0.875 | 0.125 |
|  | **207** | 0 | 0 | 0 | 0 | 0.021 | 0 | 0.125 |
|  | **208** | 0 | 0.004 | 0 | 0.014 | 0 | 0 | 0 |
|  | **210** | 0.522 | 0.051 | 0 | 0.171 | 0.021 | 0 | 0.125 |
|  | **212** | 0.044 | 0.004 | 0 | 0.014 | 0 | 0 | 0 |
|  | **214** | 0.200 | 0.013 | 0 | 0.043 | 0.042 | 0 | 0.250 |
|  | **216** | 0.044 | 0.008 | 0 | 0.029 | 0 | 0 | 0 |
|  | **218** | 0.100 | 0 | 0 | 0 | 0 | 0 | 0 |
|  | **219** | 0.089 | 0.025 | 0 | 0.086 | 0 | 0 | 0 |
|  | **228** | 0 | 0.004 | 0 | 0.014 | 0 | 0 | 0 |
|  |  |  |  |  |  |  |  |  |
| **VVIP26** | **120** | 0 | 0.526 | 0.595 | 0.344 | 0.040 | 0.025 | 0.100 |
|  | **130** | 0 | 0.004 | 0 | 0.016 | 0.360 | 0.450 | 0 |
|  | **134** | 0.111 | 0.004 | 0 | 0.016 | 0 | 0 | 0 |
|  | **140** | 0.033 | 0.013 | 0 | 0.047 | 0 | 0 | 0 |
|  | **142** | 0 | 0.004 | 0 | 0.016 | 0 | 0 | 0 |
|  | **148** | 0.011 | 0 | 0 | 0 | 0 | 0 | 0 |
|  | **152** | 0 | 0.009 | 0 | 0.031 | 0.160 | 0.125 | 0.300 |
|  | **154** | 0.189 | 0.022 | 0 | 0.078 | 0 | 0 | 0 |
|  | **156** | 0 | 0.358 | 0.405 | 0.234 | 0.020 | 0 | 0.100 |
|  | **160** | 0.289 | 0.022 | 0 | 0.078 | 0.020 | 0 | 0.100 |
|  | **162** | 0.011 | 0 | 0 | 0 | 0.380 | 0.400 | 0.300 |
|  | **166** | 0.167 | 0.039 | 0 | 0.141 | 0.020 | 0 | 0.100 |
|  | **180** | 0.189 | 0 | 0 | 0 | 0 | 0 | 0 |
|  |  |  |  |  |  |  |  |  |
| **VVMD5** | **226** | 0.289 | 0.018 | 0 | 0.059 | 0 | 0 | 0 |
|  | **228** | 0.133 | 0.049 | 0.006 | 0.147 | 0.140 | 0.100 | 0.300 |
|  | **232** | 0.133 | 0.013 | 0 | 0.044 | 0 | 0 | 0 |
|  | **234** | 0.111 | 0.009 | 0.006 | 0.015 | 0 | 0 | 0 |
|  | **236** | 0.089 | 0.009 | 0 | 0.029 | 0 | 0 | 0 |
|  | **238** | 0.111 | 0.004 | 0 | 0.015 | 0 | 0 | 0 |
|  | **240** | 0.111 | 0.031 | 0.013 | 0.074 | 0.020 | 0 | 0.100 |
|  | **246** | 0.022 | 0.438 | 0.506 | 0.279 | 0 | 0 | 0 |
|  | **248** | 0 | 0.366 | 0.436 | 0.206 | 0.020 | 0 | 0.100 |
|  | **250** | 0 | 0.027 | 0.019 | 0.044 | 0 | 0 | 0 |
|  | **252** | 0 | 0.013 | 0 | 0.044 | 0 | 0 | 0 |
|  | **254** | 0 | 0.004 | 0 | 0.015 | 0 | 0 | 0 |
|  | **255** | 0 | 0 | 0 | 0 | 0.320 | 0.300 | 0.400 |
|  | **258** | 0 | 0.004 | 0.006 | 0 | 0 | 0 | 0 |
|  | **260** | 0 | 0.004 | 0 | 0.015 | 0 | 0 | 0 |
|  | **264** | 0 | 0.009 | 0.006 | 0.015 | 0 | 0 | 0 |
|  | **265** | 0 | 0 | 0 | 0 | 0.420 | 0.500 | 0.100 |
|  | **267** | 0 | 0 | 0 | 0 | 0.080 | 0.100 | 0 |
|  |  |  |  |  |  |  |  |  |
| **VMC7f2** | **191** | 0.022 | 0 | 0 | 0 | 0 | 0 | 0 |
|  | **193** | 0 | 0 | 0 | 0 | 0.154 | 0.167 | 0.100 |
|  | **195** | 0 | 0.004 | 0 | 0.014 | 0 | 0 | 0 |
|  | **197** | 0.122 | 0.021 | 0.012 | 0.043 | 0 | 0 | 0 |
|  | **199** | 0.667 | 0.966 | 0.988 | 0.914 | 0.808 | 0.810 | 0.800 |
|  | **201** | 0.022 | 0.004 | 0 | 0.014 | 0 | 0 | 0 |
|  | **203** | 0.133 | 0.004 | 0 | 0.014 | 0.038 | 0.024 | 0.100 |
|  | **205** | 0.033 | 0 | 0 | 0 | 0 | 0 | 0 |
|  |  |  |  |  |  |  |  |  |
| **UDV108** | **190** | 0 | 0.004 | 0 | 0.015 | 0 | 0 | 0 |
|  | **204** | 0.012 | 0.263 | 0.292 | 0.191 | 0 | 0 | 0 |
|  | **206** | 0 | 0.640 | 0.708 | 0.471 | 0.060 | 0.050 | 0.100 |
|  | **210** | 0 | 0 | 0 | 0 | 0.520 | 0.550 | 0.400 |
|  | **214** | 0 | 0 | 0 | 0 | 0.080 | 0.100 | 0 |
|  | **216** | 0.024 | 0 | 0 | 0 | 0 | 0 | 0 |
|  | **218** | 0.256 | 0.038 | 0 | 0.132 | 0.020 | 0 | 0.100 |
|  | **230** | 0 | 0 | 0 | 0 | 0.020 | 0.025 | 0 |
|  | **234** | 0 | 0 | 0 | 0 | 0.120 | 0.075 | 0.300 |
|  | **238** | 0.110 | 0.008 | 0 | 0.029 | 0.020 | 0 | 0.100 |
|  | **240** | 0.012 | 0 | 0 | 0 | 0 | 0 | 0 |
|  | **242** | 0.317 | 0.030 | 0 | 0.103 | 0 | 0 | 0 |
|  | **244** | 0.098 | 0 | 0 | 0 | 0 | 0 | 0 |
|  | **248** | 0.110 | 0.013 | 0 | 0.044 | 0 | 0 | 0 |
|  | **250** | 0 | 0.004 | 0 | 0.015 | 0 | 0 | 0 |
|  | **261** | 0 | 0 | 0 | 0 | 0.120 | 0.150 | 0 |
|  | **263** | 0 | 0 | 0 | 0 | 0.020 | 0.025 | 0 |
|  | **265** | 0 | 0 | 0 | 0 | 0.020 | 0.025 | 0 |
|  | **268** | 0.012 | 0 | 0 | 0 | 0 | 0 | 0 |
|  | **278** | 0.049 | 0 | 0 | 0 | 0 | 0 | 0 |
|  |  |  |  |  |  |  |  |  |
| **VMCNG3a10** | **94** | 0.022 | 0 | 0 | 0 | 0 | 0 | 0 |
|  | **96** | 0.011 | 0.004 | 0 | 0.014 | 0 | 0 | 0 |
|  | **100** | 0.033 | 0.047 | 0.019 | 0.114 | 0 | 0 | 0 |
|  | **102** | 0 | 0.004 | 0 | 0.014 | 0 | 0 | 0 |
|  | **104** | 0.011 | 0 | 0 | 0 | 0 | 0 | 0 |
|  | **106** | 0.133 | 0.034 | 0.006 | 0.100 | 0.540 | 0.525 | 0.600 |
|  | **108** | 0.067 | 0.841 | 0.963 | 0.557 | 0.040 | 0 | 0.200 |
|  | **109** | 0 | 0.013 | 0.012 | 0.014 | 0 | 0 | 0 |
|  | **110** | 0 | 0.004 | 0 | 0.014 | 0.080 | 0.100 | 0 |
|  | **112** | 0.178 | 0.013 | 0 | 0.043 | 0.040 | 0.050 | 0 |
|  | **114** | 0.089 | 0 | 0 | 0 | 0.220 | 0.250 | 0.100 |
|  | **116** | 0.289 | 0.009 | 0 | 0.029 | 0.060 | 0.075 | 0 |
|  | **118** | 0.111 | 0.013 | 0 | 0.043 | 0.020 | 0 | 0.100 |
|  | **134** | 0.056 | 0.017 | 0 | 0.057 | 0 | 0 | 0 |
|  |  |  |  |  |  |  |  |  |
| **VMC5a10** | **140** | 0 | 0.008 | 0 | 0.029 | 0 | 0 | 0 |
|  | **141** | 0.011 | 0 | 0 | 0 | 0 | 0 | 0 |
|  | **146** | 0 | 0.029 | 0.036 | 0.014 | 0.212 | 0.238 | 0.100 |
|  | **148** | 0 | 0 | 0 | 0 | 0.135 | 0.167 | 0 |
|  | **150** | 0.466 | 0.181 | 0.143 | 0.271 | 0.019 | 0 | 0.100 |
|  | **152** | 0.205 | 0.761 | 0.815 | 0.629 | 0.577 | 0.571 | 0.600 |
|  | **154** | 0 | 0.008 | 0.006 | 0.014 | 0 | 0 | 0 |
|  | **158** | 0 | 0 | 0 | 0 | 0.019 | 0 | 0.100 |
|  | **159** | 0.205 | 0.008 | 0 | 0.029 | 0 | 0 | 0 |
|  | **161** | 0.114 | 0 | 0 | 0 | 0 | 0 | 0 |
|  | **162** | 0 | 0.004 | 0 | 0.014 | 0 | 0 | 0 |
|  | **170** | 0 | 0 | 0 | 0 | 0.019 | 0.024 | 0 |
|  | **172** | 0 | 0 | 0 | 0 | 0.019 | 0 | 0.100 |
|  |  |  |  |  |  |  |  |  |
| **VVMD7*** | **233** | 0.011 | 0.008 | 0 | 0.029 | 0 | 0 | 0 |
|  | **239** | 0.467 | 0.134 | 0.018 | 0.414 | 0.019 | 0 | 0.100 |
|  | **241** | 0 | 0.588 | 0.714 | 0.286 | 0.135 | 0.143 | 0.100 |
|  | **243** | 0.111 | 0.034 | 0 | 0.114 | 0 | 0 | 0 |
|  | **247** | 0.089 | 0 | 0 | 0 | 0 | 0 | 0 |
|  | **249** | 0.144 | 0.025 | 0 | 0.086 | 0.019 | 0 | 0.100 |
|  | **251** | 0.022 | 0 | 0 | 0 | 0 | 0 | 0 |
|  | **253** | 0.033 | 0 | 0 | 0 | 0 | 0 | 0 |
|  | **255** | 0.022 | 0.013 | 0.006 | 0.029 | 0.827 | 0.857 | 0.700 |
|  | **257** | 0.056 | 0 | 0 | 0 | 0 | 0 | 0 |
|  | **261** | 0 | 0.004 | 0 | 0.014 | 0 | 0 | 0 |
|  | **263** | 0.044 | 0 | 0 | 0 | 0 | 0 | 0 |
|  | **Null X2** | 0 | 0.193 | 0.262 | 0.029 | 0 | 0 | 0 |
|  |  |  |  |  |  |  |  |  |
| **VVMD31*** | **201** | 0 | 0 | 0 | 0 | 0.115 | 0.119 | 0.100 |
|  | **204** | 0.100 | 0 | 0 | 0 | 0 | 0 | 0 |
|  | **206** | 0.056 | 0.021 | 0 | 0.071 | 0 | 0 | 0 |
|  | **208** | 0 | 0.008 | 0 | 0.029 | 0 | 0 | 0 |
|  | **210** | 0.144 | 0.025 | 0 | 0.086 | 0 | 0 | 0 |
|  | **212** | 0.367 | 0.130 | 0 | 0.443 | 0.038 | 0 | 0.200 |
|  | **214** | 0.089 | 0.059 | 0.012 | 0.171 | 0.654 | 0.667 | 0.600 |
|  | **216** | 0.233 | 0.017 | 0 | 0.057 | 0.154 | 0.167 | 0.100 |
|  | **224** | 0.011 | 0 | 0 | 0 | 0 | 0 | 0 |
|  | **Null X2** | 0 | 0.740 | 0.988 | 0.143 | 0.038 | 0.048 | 0 |
|  |  |  |  |  |  |  |  |  |
| **VVMD32*** | **239** | 0 | 0.009 | 0 | 0.029 | 0 | 0 | 0 |
|  | **241** | 0.267 | 0.031 | 0 | 0.100 | 0 | 0 | 0 |
|  | **245** | 0 | 0 | 0 | 0 | 0.154 | 0.190 | 0 |
|  | **251** | 0.144 | 0.004 | 0 | 0.014 | 0 | 0 | 0 |
|  | **253** | 0.089 | 0.889 | 1.000 | 0.643 | 0.115 | 0 | 0.600 |
|  | **257** | 0.089 | 0.035 | 0 | 0.114 | 0.038 | 0 | 0.200 |
|  | **259** | 0.056 | 0.027 | 0 | 0.086 | 0.038 | 0 | 0.200 |
|  | **263** | 0.044 | 0 | 0 | 0 | 0 | 0 | 0 |
|  | **265** | 0.056 | 0.004 | 0 | 0.014 | 0 | 0 | 0 |
|  | **273** | 0.256 | 0 | 0 | 0 | 0 | 0 | 0 |
|  | **Null X2** | 0 | 0 | 0 | 0 | 0.654 | 0.810 | 0 |
|  |  |  |  |  |  |  |  |  |
| *At these three loci there were no results for multiple wild-collected samples. | | | | | | | | |
