# Supplementary material for: Hybridization of cultivated Vitis vinifera with wild V. californica and V. girdiana in California
Source: Ecol Evol. 2015 Nov 19;5(23):5671–84. doi: 10.1002/ece3.1797 (PMC4813103; doi:10.1002/ece3.1797)
Supplement: Supplementary file 4 — Table S3. Estimated membership coefficient and posterior probabilities of correct assignment and immigrant ancestry for 26 wild‐collected Vitis girdiana vines. [file ECE3-5-5671-s004.docx]

| **Table S3.** The estimated membership coefficient for the *Vitis. girdiana* cluster (Q_GRD_), the posterior probability of correct assignment and the posterior probability of immigrant ancestry for the 26 vines collected as wild *V*. *girdiana*. The probabilities indicate if an individual is correctly assigned to the *V. girdiana* cluster, is best assigned to another cluster or has recent ancestry in another clusters. Bold font highlights the largest portion of probability for the five hybrids, one of which is known to be a 'Mission' first generation hybrid. | | | | | | | | | | | |
| --- | --- | --- | --- | --- | --- | --- | --- | --- | --- | --- | --- |
|  |  | ***Vitis Girdiana*** | **Probability of *Vitis vinifera* assignment or ancestry** | | | |  | **Probability of *Vitis californica* assignment or ancestry** | | | |
|  | **Q_GRD_** | **Assignment Probability** | **Assignment** | **1st generation** | **2nd generation** | **3rd generation** |  | **Assignment** | **1st generation** | **2nd generation** | **3rd generation** |
| SC50 | 1.00 | 1.00 | 0 | 0 | 0 | 0 |  | 0 | 0 | 0 | 0 |
| SC51 | 1.00 | 1.00 | 0 | 0 | 0 | 0 |  | 0 | 0 | 0 | 0 |
| SC53 | 1.00 | 1.00 | 0 | 0 | 0 | 0 |  | 0 | 0 | 0 | 0 |
| SC26 | 1.00 | 1.00 | 0 | 0 | 0 | 0 |  | 0 | 0 | 0 | 0 |
| SC36 | 1.00 | 1.00 | 0 | 0 | 0 | 0 |  | 0 | 0 | 0 | 0 |
| SC52 | 1.00 | 1.00 | 0 | 0 | 0 | 0 |  | 0 | 0 | 0 | 0 |
| SC13 | 1.00 | 1.00 | 0 | 0 | 0 | 0 |  | 0 | 0 | 0 | 0 |
| SC30 | 1.00 | 1.00 | 0 | 0 | 0 | 0 |  | 0 | 0 | 0 | 0 |
| SC33 | 1.00 | 1.00 | 0 | 0 | 0 | 0 |  | 0 | 0 | 0 | 0 |
| SC38 | 1.00 | 1.00 | 0 | 0 | 0 | 0 |  | 0 | 0 | 0 | 0 |
| SC40 | 1.00 | 1.00 | 0 | 0 | 0 | 0 |  | 0 | 0 | 0 | 0 |
| ANU78 | 1.00 | 1.00 | 0 | 0 | 0 | 0 |  | 0 | 0 | 0 | 0 |
| SC39 | 1.00 | 1.00 | 0 | 0 | 0 | 0 |  | 0 | 0 | 0 | 0 |
| SC9 | 1.00 | 1.00 | 0 | 0 | 0 | 0 |  | 0 | 0 | 0 | 0 |
| SC4 | 1.00 | 1.00 | 0 | 0 | 0 | 0 |  | 0 | 0 | 0 | 0 |
| SC1 | 0.99 | 1.00 | 0 | 0 | 0 | 0 |  | 0 | 0 | 0 | 0 |
| SC2 | 0.99 | 1.00 | 0 | 0 | 0 | 0 |  | 0 | 0 | 0 | 0 |
| SC37 | 0.99 | 1.00 | 0 | 0 | 0 | 0 |  | 0 | 0 | 0 | 0 |
| SC11 | 0.99 | 1.00 | 0 | 0 | 0 | 0 |  | 0 | 0 | 0 | 0 |
| SC12 | 0.99 | 1.00 | 0 | 0 | 0 | 0 |  | 0 | 0 | 0 | 0 |
| SC3 | 0.97 | 0.97 | 0 | 0 | 0 | 0 |  | 0 | 0 | 0 | 0.03 |
| ANU25 | 0.81 | 0.06 | 0 | 0 | **0.51** | 0.44 |  | 0 | 0 | 0 | 0 |
| SC14 | 0.67 | 0 | 0 | 0 | **0.93** | 0.07 |  | 0 | 0 | 0 | 0 |
| SC19 | 0.64 | 0 | 0 | 0 | 0 | 0 |  | 0 | 0 | **0.98** | 0.02 |
| SC27 | 0.63 | 0 | 0.10 | 0 | 0 | 0 |  | 0.02 | 0 | **0.69** | 0.19 |
| SC42* | 0.52 | 0 | 0.33 | **0.51** | 0.16 | 0 |  | 0.00 | 0 | 0 | 0 |
| *This vine is a 'Mission' F1 hybrid. | | | | | | | | | | | |
